# Is the Isthmus Location an Additional Risk Factor for Indeterminate Thyroid Nodules? Case Report and Review of the Literature

**DOI:** 10.3389/fendo.2018.00750

**Published:** 2018-12-13

**Authors:** Gilda Pontieri, Francesca Urselli, Livia Peschi, Alessia Liccardi, Anna Rita Ruggiero, Emilia Vergara, Claudio Bellevicine, Giancarlo Troncone, Maurizio De Palma, Bernadette Biondi

**Affiliations:** ^1^Department of Clinical Medicine and Surgery University of Naples Federico II, Naples, Italy; ^2^Dipartimento Assistenziale Integrato di Oncoematologia, Diagnostica per Immagini e Morfologica e Medicina Legale A.O.U. Federico II, Naples, Italy; ^3^Department of Public Health University of Naples Federico II, Naples, Italy; ^4^Dipartimento Chirurgico Generale e Polispecialistico, Chirurgia 2 AORN Cardarelli, Naples, Italy

**Keywords:** indeterminate thyroid lesion, isthmus nodule, multifocality, capsular invasion, extrathyroidal extension, lymph nodes metastasis, total thyroidectomy, isthmusectomy

## Abstract

**Background:** The management of indeterminate thyroid lesions is controversial. The American Thyroid Association (ATA) guidelines suggest a conservative approach for low risk indeterminate thyroid lesions (TIR3A).

**Case Report:** We report a clinical case of a young girl who had TIR3A in a thyroid nodule located in the isthmus. After considering clinical and ultrasound (US) risk factors, we assessed literature data and guidelines to plan the extension of surgery. We found several studies supporting that the isthmus malignant lesions were associated with a higher rate of multifocality, capsular invasion, extrathyroidal extension, and central lymph node (LN) metastases. These data could predict a more aggressive behavior and a poor prognosis of the isthmus thyroid cancer compared to differentiated thyroid cancer, originating in the thyroid lobes. On the basis of these literature data and considering the familial risk for thyroid cancer of our patient, we decided to perform a total thyroidectomy. The histological examination revealed a follicular variant of papillary carcinoma located in the isthmus with capsular invasion.

**Conclusion:** The isthmus location could be an additional risk factor to consider for a correct surgical approach in indeterminate thyroid lesions and thyroid cancer at fine-needle aspiration (FNA). We suggest that a careful ultrasonography should be carried out in patients with isthmus nodules. Total thyroidectomy should be performed in aggressive nodular disease. Prospective studies are needed to establish the best treatment for these lesions.

## Introduction

The management of the indeterminate thyroid nodule in clinical practice is still a matter of debate. The American Thyroid Association (ATA) guidelines suggest a conservative strategy for TIR3A (low risk indeterminate lesions according to the Bethesda System) by repeating the fine-needle aspiration (FNA) and performing molecular tests (when available) (1). If the repetition of FNA cytology confirms a TIR3A, a surveillance program with a clinical and ultrasound (US) follow-up or surgical excision for a definitive diagnosis may be performed depending on the clinical risk factors, sonographic features, patient preference, and results of the molecular tests (1). On the other hand, surgery is recommended as a first choice treatment for high risk indeterminate lesions (TIR3B) (1). This approach is justified by the higher risk of malignancy in TIR3B vs. TIR3A, although this risk differ in the international subclassifications of indeterminate thyroid nodules (1–3). The risk of malignancy was < 10 and 15–30%, respectively for TIR3A and TIR3B in a recent Italian consensus for the reporting of thyroid cytology (ICCRTC) (4). A meta-analysis including six retrospective studies confirmed that this classification allows clinicians to distinguish between low and high risk malignancy in indeterminate lesions (5). Lobectomy is recommended as the initial approach when surgery is indicated in patients with a solitary indeterminate thyroid nodule. Total thyroidectomy may be preferred in patients with large nodules (>4 cm), indeterminate nodules with sonographic or cytological findings suspicious for malignancy, positivity for specific mutations associated with thyroid carcinoma, familial thyroid cancer, and history of radiation exposure (1, 2).

The incidence of thyroid carcinoma in the isthmus ranges between 3 and 9.2% (6, 7).

We report the clinical case of a young girl with an isolated indeterminate thyroid nodule (TIR3A) located in the isthmus. No standard treatment is recommended when thyroid lesions result as TIR3A or papillary thyroid cancer (PTC) in the isthmus. Current guidelines do not support the surgical management of thyroid cancer with procedures other than thyroid lobectomy, near-total, and total thyroidectomy. Isthmusectomy is not reported in the ATA guidelines as an appropriate surgical procedure for differentiated thyroid cancer. International guidelines have not formulated specific recommendations for a correct surgical approach of the isthmus nodule, neither for carcinoma nor for indeterminate nodules.

Therefore, we performed a review of the literature to assess the aggressiveness and prognosis associated with these lesions.

## Case Report

**Figure 1:**
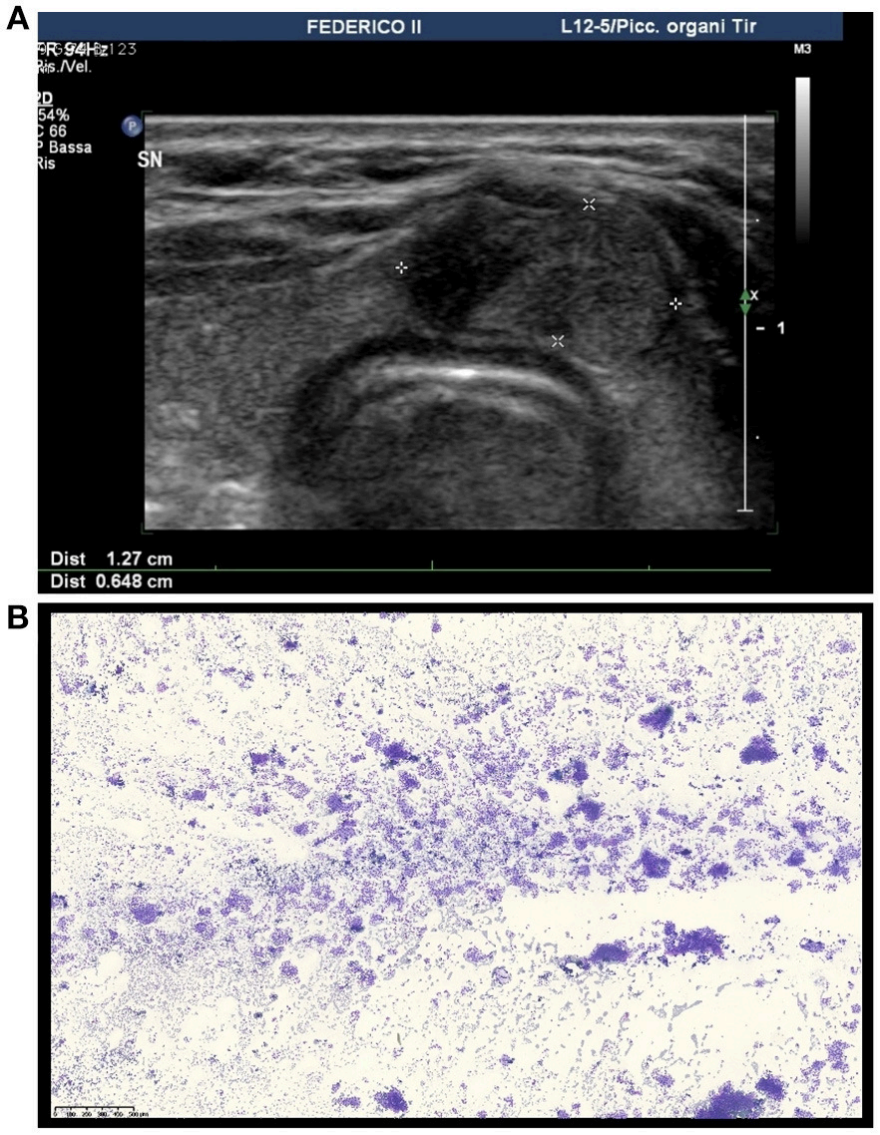
Ultrasound and cytological features of the thyroid nodule in our patient. **(A)** Ultrasound image showing an isoechoic solid nodule with a hypoechoic cranial component with blurred margins located in the isthmus of the thyroid. **(B)** Medium power magnification showing a hypercellular smear featuring thyrocytes arranged in microfollicular structures (DiffQuik staining, 100X).

An 18 year old woman was referred to our outpatient clinic of Endocrinology, University-Hospital of Naples Federico II because of hypothyroidism due to Hashimoto's thyroiditis. Blood samples showed high levels of thyroperoxidase and thyroglobulin antibodies and normal calcitonin serum levels. The patient was euthyroid with normal serum levels of thyroid-stimulating hormone (TSH), free triiodothyronine (FT3), and free thyroxine (FT4) during replacement therapy with L-T4. At physical examination, a palpable nodule of ~2 cm in size was detected in the isthmus of the thyroid. There were no palpable cervical lymph-nodes. An US evaluation confirmed an isolated lesion located in the isthmus, showing an isoechoic solid nodule with smooth margins; its size was 18 × 13 × 6 mm with intra and perilesional vascularity (Figure 1A). Therefore, a FNA was performed and cytological results revealed a TIR3A lesion. The cytological specimen showed an increased cellularity with some microfollicular structures in the background of scant colloid (Figure 1B). Thus, we assessed the risk factors associated with the isolated TIR 3A nodule of our patient. According to the ATA guidelines we repeated the FNA which confirmed the same result (TIR3A). The second US (after 6 months) showed that there were no clear signs suggesting malignancy such as microcalcifications or taller than wide-shaped nodules. However, we found a small hypoechoic cranial component in the nodule with blurred margins and elastography revealed an increased stiffness in this cranial component. No nodules were detected in the contralateral lobes by US; cervical lymph nodes were normal. Among the possible risk factors, our patient referred a familial history of thyroid cancer. Her mother was submitted to total thyroidectomy for a follicular variant of PTC twenty years ago; our subsequent evaluation showed that she was disease free at the moment.

On this basis, we decided that a surgical treatment was indicated for our patient and assessed the risk/benefit of total thyroidectomy vs. isthmusectomy.

## Discussion

We assessed literature data on the relationship between the site of the thyroid lesions and the complications associated with the extension of surgery to decide whether an isthmusectomy was a sufficient surgical option in our patient or if a more extensive surgery was needed. A few studies have reported the results of isthmusectomy in thyroid cancer (7–9) and examined the postoperative complications of total thyroidectomy vs. isthmusectomy or isthmus-lobectomy. Some reports have suggested that when PTC is confined in the isthmus, thyroid isthmusectomy could be the first surgical choice because of the reduced risk of damaging the recurrent laryngeal nerve and parathyroid glands (10, 11). In fact, this surgical option allows surgeons to maintain the dissection on the anterior surface of the trachea, avoiding the exploration of the trachesophageal groove or the identification of the recurrent laryngeal nerve. Moreover, the parathyroid glands are not exposed during this surgical procedure. One prospective study including nine patients with indeterminate thyroid nodules, or nodules suspicious of malignancy located in the isthmus or pyramidal lobe, reported that isthmusectomy was a safe procedure in patients with a maximum diameter of 30 mm (12). On the other hand, some reports have found no significant difference in recurrent laryngeal nerve injury between lobectomy and total thyroidectomy in a group of patients with microcarcinoma, although the risk of permanent hypoparathyroidism was higher in the total thyroidectomized group than in the lobectomized group (1.7 vs. 0%, respectively) (8).

Regarding the prognosis of thyroid cancer, no significant difference for recurrent disease between lobectomy and total thyroidectomy was detected in patients with low and intermediate risk of structural disease recurrence (13, 14). One large retrospective study from Memorial Sloan Kettering Cancer Center, from a database of 1,810 patients, assessed the outcome of patients treated with thyroid isthmusectomy alone for localized well-differentiated thyroid cancer during a 20-year period. The authors reported that only 19 patients with PTC (1%) were suitable for isthmusectomy, between 1986 and 2005, because of an isolated lesion of the thyroid isthmus without evidence of extraglandular spread. The regional and distant recurrence-free survival were 100% in this study (10). All of these literature data could support the concept that isthmusectomy could be suitable in patients with solitary nodules, confined to the isthmus without evidence of extraglandular extension to avoid the dissection of the recurrent laringeal nerve and parathyroid glands.

We also carried out a review of the literature to assess if the isthmus location could represent an additional risk factor to plan the extension of surgery. Therefore, we analyzed the risk of multifocality, capsular invasion, extrathyroidal extension, and lymph node (LN) metastasis as pathological features associated with the isthmus cancer compared to the carcinoma originating in the thyroid lobe (Table 1). We found several studies reporting that thyroid malignant lesions located in the isthmus were more aggressive and associated with poor prognosis (6, 15, 22–24).

**Table 1:** Literature data of main features of isthmus vs. non isthmus thyroid cancer.

**Authors**	**Lee et al. (15)**	**Hahn et al. (16)**	**Karatzas et al. (17)**	**Goldforb et al. (18)**	**Lee et al. (6)**	**Song et al. (19)**	**Wang et al. (20)**	**Xiang et al. (21)**
No. of patients	190	2,623 (144 analyzed in the study)	575	281	1,973	194	3,577	949
Patients with isthmus DTC	**7.3%**	**2.2%** (48 analyzed in the study)	**9.3%**	**4.2%**	**9.2%**	**45**	**2%**	**7.3%**
Surgical procedure in isthmus DTC vs. in non isthmus DTC	**TT+CDN vs**. TT+CDN	**81.3% TT+CND 18.7% TT+CND+LND vs**. 85.4% TT+CND 14.6% TT+CND+LND	TT+CND^*1^ **vs**. TT+CND*^1^	TT ± CND+LND^*2^ **vs**. –	**90.6% TT+CND ipsilateral 9.4% TT+CND+LND vs**. 81.8% TT+CND ipsilateral 18.2% TT+CND+LND	**86.7% TT+CND 13.3% TT+ CND+ LND vs**. 83.3% TT+CND 16.7% TT+CND+LND	**TT+ CND vs**. –	SubTT+CND+LND^*2^ **vs**. Loboisthmusectomy or TT+CND+LND*^2^
Patients with multifocality in isthmus DTC vs. in non isthmus DTC	**64.3 vs**. 40.3%	**54.2 vs**. 45.8%	**51.9 vs**. 35.7%	**67 vs**. –	**48.6 vs**. 39.8%	–	–	–
Patients with capsular invasion in isthmus DTC vs. in non isthmus DTC	–	–	**25.9 vs**. 22.1%	**33 vs**. –	**70.2 vs**. 60.8%	**46.7 vs**. 4.4%	–	–
Patients with ETE in isthmus DTC vs. in non isthmus DTC	**100 vs**. 54%	**83.3 vs**. 65.6%	–	–	–	–	**11% vs**. –	–
Patients with CLN mts in isthmus DTC vs. in non isthmus DTC	**71.4 vs**. 44.6%	**68.8 vs**. 58.3%	**29.6 vs**. 16.3%	**50% vs**. –	**40.3 vs**. 42.1%	**71.1 vs**. 40.3%	**46.6% vs**. –	**44 vs**. 28.2%
Patients with LLN mts in isthmus vs. in non isthmus DTC	**14.3 vs**. 11.9%	**16.7 vs**. 14.6%		8% **vs**. –	**9.4 vs**. 18.2%		–	**4 vs**. 4.6%

DTC, differentiated thyroid cancer; TT, total thyroidectomy; ETE, extrathyroidal extension; CLN, central lymph nodes; LLN, lateral lymph nodes; CND, central lymph node dissection; LND, lateral lymph node dissection; mts, metastasis;

*1 if mts detected by FNA or palpation;

*2 *if mts detected by US or FNA. Bold values correspond to patients with isthmus DTC*.

Literature data reported a higher rate of multifocality in the isthmic PTC compared to cancers located in other parts of the thyroid gland (18). In fact, since the isthmus is a very small portion of the parenchyma and its location is in the center of the thyroid, it is reasonable that isthmus cancer could spread into one of the two lobes. Moreover, the isthmus thickness is 2–6 mm, therefore, capsular invasion and extrathyroidal extension could be more frequent in the isthmus cancer than in those originating in the thyroid lobes (16). Literature data confirmed that capsular invasion and extrathyroidal extension were independent of tumor size in the isthmus cancers because these findings were also frequent in the isthmus microcarcinoma (17).

We found several studies also demonstrating a higher frequency of LN metastases in thyroid cancer located in the isthmus compared to non-isthmus carcinoma (19, 21). Thyroid isthmus has a different lymphatic drainage compared to the thyroid lobe and in particular, lymphatic isthmic vessels usually drain into the prelaryngeal, pretracheal, and paratracheal LN. Prelayngeal LN are also called Delphian, from “the Oracle of Delphi,” a greek legend, predicting an unfavorable prognosis (22). We also found studies reporting a more frequent involvement of the central compartment in patients with isthmus carcinoma, probably due to the particular isthmus lymphatic drainage. Interestingly, some authors (17) showed that there was no statistically significant difference between LN metastasis with isthmus cancer <10 mm and >10 mm suggesting that the isthmus location could be a risk factor for central LN involvement, regardless of tumor size. Therefore, many authors considered total thyroidectomy with bilateral central LN compartment dissection as an appropriate surgical approach for patients with isthmic PTC, due to the high rate of bilateral central LN metastasis. (6, 25, 20). Moreover, a limited experience from an Italian group suggest the possible effectiveness of radioiodine (RAI) ablation in patients with isthmic thyroid cancer (26).

These findings could suggest that the isthmus location could be an additional risk factor for indeterminate thyroid nodules and, therefore, total thyroidectomy could represent the most appropriate therapeutic strategy in patients with thyroid cancer located in the isthmus because of its more frequent multifocality, capsular invasion, extrathyroidal extension, and LN metastasis.

However, our accurate analysis found important limits of the literature studies on the isthmus nodules because the majority of the available data are retrospective and heterogeneous differing in terms of patient's age and sex, tumor size, and extension of surgery. Moreover, a small sample of malignant lesions have been included in these reports.

Our young patient had a familial history of thyroid cancer. The ATA guidelines recommend that total thyroidectomy may be the preferred treatment of indeterminate thyroid lesions in presence of a familial history of thyroid carcinoma because of the increased risk of malignancy (1). Several authors demonstrated that familial PTC is a distinct and isolated entity rather than a casual association of the same disease in a family. In fact, some studies reported an increased incidence of PTC when parents or siblings were affected by PTC with a particular risk among sisters and, an appearance, at an earlier age in the second generation (27, 28). Moreover, a “genetic anticipation” of PTC was found, even in the presence of only two family members affected by thyroid malignancy. Indeed, tumors in the second generation of the family were diagnosed at a younger age and were more aggressive, showing a multifocality and a higher rate of local and distant metastasis (29).

Therefore, considering the familial history, US risk factors and the literature regarding the isthmus cancer we decided to perform a total thyroidectomy in our patient. The surgical treatment was planned at the surgical center of our hospital (where a high volume of thyroidectomies per year is performed).

The histological examination revealed a follicular variant of papillary carcinoma. The tumor was located in the isthmus with capsular invasion; no additional LN were sampled. The immunohistochemical panel was positive for Galectin-3, HBME-1, and negative for CD56. According to the American Joint Committee on Cancer (AJCC) 8th edition, it was classified as T1bNx. One year after treatment, our patient is now disease free with undetectable levels of thyroglobulin and thyroglobulin antibodies and without any complications from surgery, although we did not perform RAI ablation. It is important to emphasize that, although a recent meta-analysis reported an association between HT and PTC, thyroid cancer in this setting was associated with a better prognosis, for the earlier discover (30).

## Concluding Remarks

We suggest that a careful US should be carried out in patients with isthmic nodules to accurately assess the dimensions of the nodules, the involvement of the thyroid lobes, signs of capsular invasion and /or extrathyroidal extension and characteristics of LNs. According to the literature results, an isthmusectomy could be the procedure of choice in patients with isolated isthmus lesion <30 mm without evidence of multifocality, extraglandular spread or LN involvement. Total thyroidectomy could be performed in invasive nodular disease, molecular tests positive for mutations that are associated with an aggressive behavior and in patients with familial thyroid cancer or history of radiation exposure.

Prospective studies are needed to establish the best treatment for isthmus indeterminate thyroid nodules or cancer.

## Author Contributions

All authors listed have made a substantial, direct and intellectual contribution to the work, and approved it for publication.

### Conflict of Interest Statement

The authors declare that the research was conducted in the absence of any commercial or financial relationships that could be construed as a potential conflict of interest.
